# Embedding a patchwork text model to facilitate meaningful reflection within a medical leadership curriculum

**DOI:** 10.5116/ijme.580b.2702

**Published:** 2016-11-17

**Authors:** Charlotte Moen, Patricia Prescott

**Affiliations:** 1 Post Graduate Professional Education, Faculty of Health & Social Care, Edge Hill University, UK

**Keywords:** Patchwork Text Model, meaningful reflection, medical leadership, curriculum

## Introduction

In the UK following the Francis report there has been a focus on clinical leadership and medical engagement as it is recognised this is essential to improving patient care, safety, organisational performance and innovation.^1^ This has resulted in the development of competency frameworks, the inclusion of clinical leadership within medical education and leadership and management standards for all doctors.^2^^-^^4^

Reflection is key to good medical practice.^2^^-^^6^ A reflective outlook raises consciousness, prompts movement from the routine of practice to its critical appraisal and drives change.  It is central to appraisal and medical revalidation.^5^^,^^6^^,^^8^ Revalidation is the process by which all UK doctors demonstrate to the General Medical Council, though annual appraisals, they are up-to-date and fit to practise.^8^ In order to provide this evidence, UK doctors complete a National Health Service reflective portfolio. The reflections are structured using the headings: Description, Feelings, Evaluation, Analysis, Conclusion and Action Plan.  For example the catalyst for reflection for an anaesthetic trainee may be performing an epidural, whilst observed by the consultant, who then ‘signs off’ for that procedure.  However the current process has been perceived as a ‘tick box’ exercise.^5^^-^^7^ In order for reflection to become *meaningful* it is critical, to develop a curriculum where reflection is meaningful, authentic and tailored to the individual. 

### Meaningful reflection within a medical context

Meaningful reflection occurs where reflection transcends the level of the action, to the deep exploration of underlying beliefs, attitudes and behaviours,^5^^,^^6^ in other words the scrutiny of personal paradigms. The key to learning from significant experiences is  the movement from the narrative, surface approach to the deeper interrogation of learning, as well as developing self-awareness in order to identify development needs.^6^ Additionally, appreciating differing opinions, for example those of peers, patients and supervisors encourages the development of new insight and the transformation of perspective. 

There are multiple perceived barriers to written reflection. Medical practitioners may not value written reflection, if it is a requirement.^5^^-^^7^ There is a perception of the time consuming nature of reflection, the reluctance to question one’s own practice and uncertainty of expectations.^6^^,^^7^ All of which might lead to a default, shallow engagement with reflection meeting threshold requirements, without commitment to the process.

It is apparent reflection is essential to a doctor’s personal development as well as to enhancing patient care. However it is clear there are some complex issues; whilst verbal reflection is acknowledged to be natural, written reflection, in an academic context at master’s level can be a significant challenge.

### A patchwork text approach

A Patchwork Text approach (PWT)^9^ is defined as:

 “... a variety of small sections, each of which is complete in itself, and that the overall unity of these component sections, although planned in advance, is finalised retrospectively, when they are ‘stitched together’. Thus a ‘patchwork text’ assignment ... is gradually assembled ... and consists of a sequence of fairly short pieces of writing”.^10^

The PWT approach promotes critical understanding particularly where insights into personal paradigms are a significant aspect of the learning process. There are several different designs however within each approach there are common elements that adhere to fundamental objectives: (a) continuous learning, (b) deep learning, (c) integrated understanding of a topic and (d) meta-cognitive self-reflection on the learning journey.^9^ Based on these objectives, a core set of elements (multiple assessment tasks; pacing of tasks and the integration of work into a comprehensive whole) together with a range of optional elements are identified.

### Application of a patchwork text approach to a medical curriculum

Doctors are required to develop their self-awareness, to understand the impact of self on others and to be critical in their thinking, in order to identify their personal strengths and development needs.^2^^,^^4^ The two key challenges are to encourage *meaningful *as opposed to a tick box approach to reflection and the development of master’s level writing. We suggest the solution is a leadership self-awareness curriculum based on an adapted PWT approach.  Below we outline this approach, applied to a master's level medical leadership module undertaken by senior trainees, in which we have incorporated all of the core elements plus most of the optional elements of the PWT approach.^9^

A Reflective Journal, with templates to be adapted or adopted, is provided to record meaningful reflections. This can include reflection on: self-awareness tool results (e.g., Emotional Intelligence; JTI Personality Type; Team Role; Resilience); benchmarking against medical standards;^2^^,^^4^ significant events within own context of practice (e.g., critical incident, service re-design, new procedure); 360 feedback; clinical audit; feedback from supervisors and patients. The reflective patches gradually develop as new self-assessment tools are explored and learning emerges. Trainees are encouraged to share their reflective patches both in the classroom setting and with ‘critical friends’. Thus the Reflective Journal scaffolds the progression from descriptive reflection towards critical reflection.

A formative assessment is integrated into the module, based on one reflective patch of the trainee’s choice (e.g., a reflection on how their personality type impacts on colleagues or how their leadership style impacts on their team/culture). The aim is two-fold; firstly to provide feedback on their academic writing, to *feed-forward* to their summative assessment, and secondly to explain how this one patch can be grown into the final assessment. Trainees choose which patches they ‘stitch’ together into their summative assessment. This is note-worthy as it facilitates a personalised approach to assessment. Although the trainees are exposed to many self-awareness tools/vehicles for reflection, they are able to explore in-depth the patches/events significant to self. The summative assessment comprises a reflective integrated portfolio that incorporates: (a) personal learning in relation to their leadership strengths and challenges, (b) insight into their leadership paradigm by considering what has shaped and influenced their perception of leaders and leadership, (c) identification of own values/behaviours and consideration of their alignment with NHS values/standards^2^^-^^4^ (d) integration of literature and key policies/drivers for change  (e) recognition of  personal leadership goals.

The process of the development of the raw reflective patches (the significant incident), through to the meaningful synthesis of learning and insight, which makes cogent connections between the past, present and future, is what makes this assessment strategy meaningful.  It prompts the interrogation of both thinking *and* feeling, exploring differing reflective perspectives, appreciating how it might *feel* to inhabit someone else’s shoes.

The assessment is built on student choice and as such is bespoke, tailored to individual needs. Flexibility is also a feature in relation to time-management; the ‘bite size’ approach encourages reflection over short time frames so the assignment can be written in stages and because the focus is self-development, the context is both personally meaningful and context specific.

It is anticipated the long term benefits for trainees will be, that they continue with this structured approach and their Reflective Journal will become a life-long tool. This strategy would prepare trainees for their annual appraisal and re-validation in relation to leadership and management competencies.

## Conclusions

Patchwork Texts is an innovative approach that supports a constructively aligned curriculum. A personal Reflective Journal facilitates doctors to be honest, self-aware and authentic in their choice of significant reflective catalysts. We suggest PWT combines meaningful reflection in and on practice, significant event analysis, and assessment *for* and *of* learning. An integrated formative assessment serves to feed-forward to the summative work and supports the development of master’s level critical reflection.  This model appears to offer an approach fit for both purpose and practice.

### Acknowledgements

We would like to thank Dr Margaret Bamforth former Associate Post Graduate Dean at Health Education England (North West) for her constructive comments on our paper. We would also like to thank Health Education England (North West) for their permission to publish this paper. Finally thanks to our colleagues at the Royal College of Physicians. The medical leadership module referred to within the article was commissioned by Health Education England (North West). 

### Conflict of interest

The authors declare that they have no conflict of interest.
